# C-reactive protein (CRP)/albumin-to-globulin ratio (AGR) is a valuable test for diagnosing periprosthetic joint infection: a single-center retrospective study

**DOI:** 10.1186/s10195-022-00657-4

**Published:** 2022-08-01

**Authors:** Hao Wu, Liping Pan, Zhichao Meng, Heng Liu, Xin Yang, Yongping Cao

**Affiliations:** Department of Orthopedics, Peking University First Hospital, No. 8 Xishiku Street, XiCheng District, Beijing, 100034 People’s Republic of China

**Keywords:** Periprosthetic joint infection, Diagnostic value, C-reactive protein/albumin-to-globulin ratio, Serum biomarker

## Abstract

**Background:**

The diagnosis of periprosthetic joint infection (PJI) is challenging for clinicians, and the commonly used methods are too complicated and expensive for many clinical practices. The neutrophil-to-lymphocyte ratio (NLR), the platelet-to-lymphocyte ratio (PLR), the platelet–to-mean-platelet-volume ratio (PVR), globulin (GLB), the albumin-to-globulin ratio (AGR), and the C-reactive protein (CRP)/AGR ratio are simple biomarkers for infection and can be easily determined from routine blood tests. Due to their low cost and ready availability in clinical practice, many clinicians have considered the diagnostic value of these biomarkers for PJI. The aim of our study is to determine the value of NLR, PLR, PVR, GLB, AGR, and CRP/AGR for the diagnosis of PJI.

**Materials and methods:**

One hundred sixty-four patients who received revision surgery after total knee or total hip replacements were enrolled, 47 in a PJI group and 117 in an aseptic failure group. Receiver operating characteristic (ROC) analysis was used to evaluate the performance of NLR, PLR, PVR, GLB, AGR, and CRP/AGR for the diagnosis of PJI, and their performance levels were then compared with those of CRP and the erythrocyte sedimentation rate (ESR).

**Results:**

The levels of all tested biomarkers were significantly higher in patients with PJI (all *P* < 0.05). ROC analysis showed that CRP/AGR performed best in diagnosing PJI, with an area under curve (AUC) value of 0.902, and the AUCs of NLR (0.740), PLR (0.721), PVR (0.668), GLB (0.719), and AGR (0.767) were all lower than those for CRP (0.896) and ESR (0.829).

**Conclusion:**

CRP/AGR was a valuable test for diagnosing PJI, but other novel biomarkers had only limited diagnostic value.

**Level of Evidence:**

Level III

## Introduction

Periprosthetic joint infection (PJI) is a very serious complication that can occur after total knee arthroplasty (TKA) and total hip arthroplasty (THA) [1]. The incidence of PJI is about 0.5 to 2.5% after primary TKA or THA, and there are approximately 1.5 infections per 1000 person-years [2–5]. PJI has a negative impact on joint mobility and patient quality of life, causes significant morbidity, and accounts for substantial health-care expenditures. The annual cost of revision operations following PJI in the US was $566 million in 2009 and will increase to $1.62 billion by 2020 [5]. However, surgeons still have difficulty in diagnosing PJI due to the lack of gold standard diagnostic criteria, and this can delay implementation of an appropriate treatment plan.

The current Musculoskeletal Infection Society (MSIS) criteria are widely used for diagnosing PJI. These criteria consider the results from blood and synovial fluid tests, a clinical examination, intraoperative histology, and cultures [6]. Although the use of these criteria significantly improves the accuracy of PJI diagnosis, many of the procedures are time-consuming, resource intensive, and invasive. Therefore, some clinicians have examined the use of inflammatory biomarkers for the timely diagnosis of PJI before revision operations. Miyamae et al. found that the level of α-defensin in synovial fluid could be used to highly accurately diagnose PJI, presenting a sensitivity of 93% and a specificity of 100% [7]. Other studies reported that synovial leukocyte esterase [8, 9] and serum fibrinogen and D-dimer [10–12] had value in the diagnosis of PJI. However, due to technical difficulties and the high cost associated with measuring these parameters, primary hospitals do not commonly perform these measurements.

Recent studies considered the use of certain serum inflammatory biomarkers obtained from routine blood examinations for the diagnosis of PJI, due to their low cost and ready availability in clinical practice. These include the mean platelet volume ratio (PVR), the neutrophil-to-lymphocyte ratio (NLR), and the platelet-to-lymphocyte ratio (PLR). For example, Paziuk et al. found that the PVR was associated with PJI (optimal cutoff: 31.70), and that using PVR with C-reactive protein (CRP) and the erythrocyte sedimentation rate (ESR) significantly improved the diagnostic accuracy of PJI compared with traditional biomarkers [13]. A recent study by Yu et al. showed that the NLR had a higher accuracy for the early diagnosis of PJI than CRP and the ESR, and receiver operating characteristic (ROC) analysis of them indicated that the area under the curve (AUC) for NLR was 0.802 [14]. Tirumala et al. found that the PLR and PVR significantly improved the accuracy of PJI diagnosis when combined with traditional serum biomarkers and joint aspirate results, with a sensitivity and specificity above 97% [15]. However, a recent study by Sigmund et al. had conflicting results in that NLR (AUC: 0.68) and PVR (AUC: 0.62) showed inferior performance in the diagnosis of PJI compared with traditional biomarkers. They therefore suggested that these markers should not be used alone for the diagnosis of PJI [16]. In addition, recent studies also investigated the levels and composition of serum albumin (ALB) and globulin (GLB) as promising biomarkers for PJI diagnosis [17, 18]. Wang et al. demonstrated that globulin (GLB) and the albumin-to-globulin ratio (AGR) had a good capacity to diagnose PJI, with an AUC greater than 0.8 [17]. Research by Shang et al. showed that AGR had good diagnostic value for PJI and could also be used to predict negative culture results and the timing of second-stage reimplantation [18].

Despite the potential diagnostic value of these novel biomarkers for PJI, the number of studies on this topic is still limited. Therefore, we designed the present single-center retrospective study to evaluate the performance of novel biomarkers (NLR, PLR, PVR, GLB, AGR, and CRP/AGR) for the diagnosis of PJI and to compare their diagnostic performance with two traditional biomarkers, ESR and CRP.

## Materials and methods

**Fig. 1:**
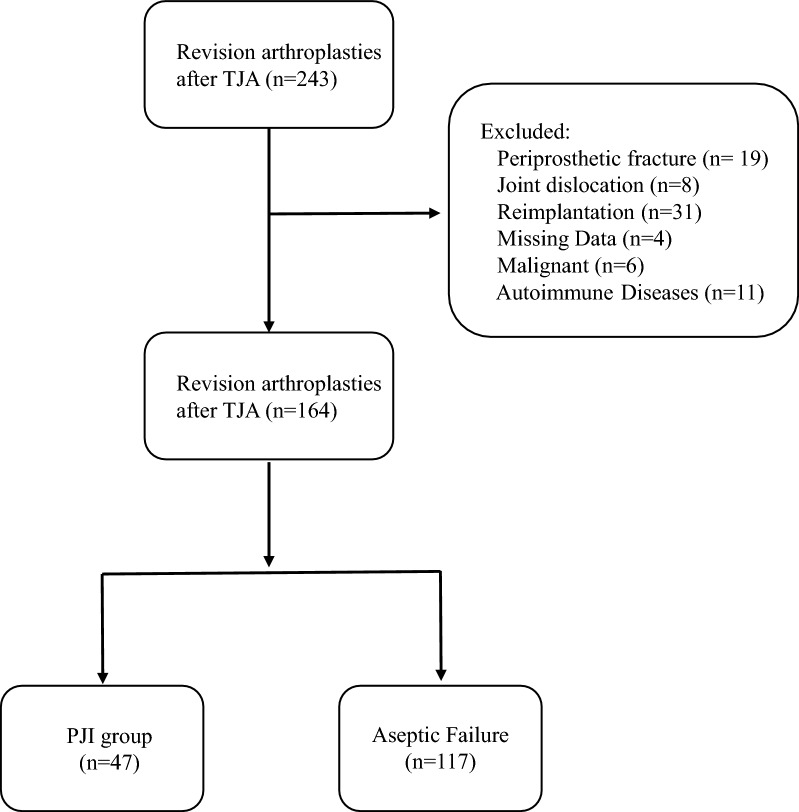
Flowchart of patient inclusion in our study

This retrospective study was approved by the Institutional Review Board (IRB) of our hospital (no. 2020-064). The records of patients who received revision surgery after TKA or THA at our institution from January 2001 to December 2019 were obtained from the electronic medical record system and reviewed. According to the MSIS criteria for PJI diagnosis (Table 1), the enrolled patients were carefully reviewed and divided into two groups: a PJI group and an aseptic revision group [6]. Because it was difficult to determine the presence of persistent infections, patients who received second-stage reimplantation were excluded. The other exclusion criteria were: diagnosis of a periprosthetic fracture or joint dislocation; missing critical data (including ESR, CRP, NLR, PVR, PLR, GLB, and AGR); presence of an autoimmune disease, such as rheumatoid arthritis or ankylosing spondylitis; and history of a malignant tumor. Finally, 164 patients were enrolled, 47 in the PJI group and 117 in the aseptic revision group (Fig. 1).

**Table 1:** MSIS criteria^a^

Major criteria	1) Two positive periprosthetic cultures with phenotypically identical organisms 2) A sinus tract communicating with the joint
Minor criteria	1) Elevated serum C-reactive protein (> 10 mg/L) *and* erythrocyte sedimentation rate (> 30 mm/h) 2) Elevated synovial fluid white blood cell count (> 3000 cells/mL) *or* change on a leukocyte esterase test strip (+ or + +) 3) Elevated synovial fluid polymorphonuclear neutrophil percentage (> 80%) 4) Positive histological analysis of periprosthetic tissue (> 5 neutrophils/HPF) based on the examination of 5 HPFs 5) A single positive culture

^a^According to the MSIS criteria, PJI is diagnosed when a patient has one of the two major criteria* or* three of the five minor criteria

All venous blood samples were collected routinely by nurses on the first or second day of admission and sent to the clinical laboratory of our hospital for testing. Joint aspiration for synovial fluid testing and the histological analysis of periprosthetic tissues was performed as needed. The periprosthetic tissues or synovial fluid collected intraoperatively were sent for aerobic and anaerobic culture.

The basic information on patients, such as age, gender, body mass index (BMI), diagnosis, treatment, comorbidities, and the results for preoperative serum biomarkers (ESR, CRP, NLR, PLR, PVR, GLB, AGR, and CRP/AGR) were collected from the electronic medical records. Test results related to PJI diagnosis, such as those from joint aspirations, the sinus tract, histological analysis, and pathogen culture findings, were also collected from these records.

### Statistical analysis

Quantitative data are expressed as means ± standard deviations, and categorical data as frequencies and ratios. The independent-samples *t*-test and the chi-squared test were used to compare the characteristics of the PJI and aseptic failure groups. Continuous variables were compared using the Mann–Whitney test and categorical variables using the chi-squared test. The diagnostic performance levels of the different biomarkers were compared based on the results of ROC analysis, calculating AUC, sensitivity, specificity, the positive predictive value (PPV), and the negative predictive value (NPV). The diagnostic value was defined based on the AUC value as either excellent (0.900–1.000), good (0.800–0.899), fair (0.700–0.799), poor (0.600–0.699), or absent (no discriminatory capacity; 0.500–0.599). The optimal threshold value for each biomarker was determined using the Youden J index. All statistical analyses were performed using MedCalc statistical software version 20 (Ostend, Belgium), and the figures were drawn using GraphPad Prism version 9 (San Diego, CA). The threshold for statistical significance was 0.05 for all analyses.

## Results

**Fig. 2:**
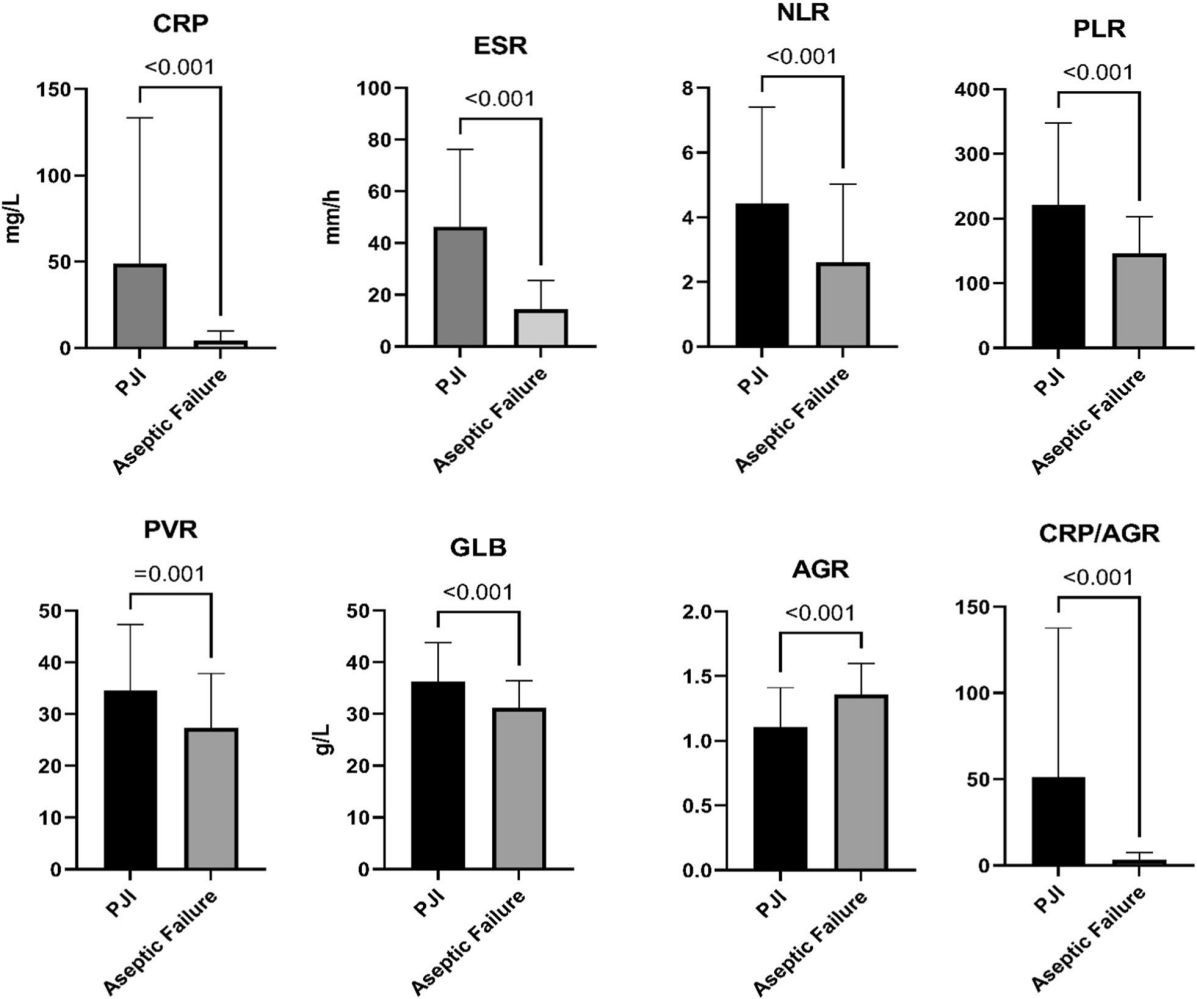
Comparison of all biomarkers between the PJI group and the aseptic failure group

We retrospectively examined 164 patients who received revision surgery after TKA or THA, and used the MSIS criteria to assign 47 patients to the PJI group and 117 patients to the aseptic revision group [6]. These two groups did not show any significant differences in BMI and gender, although the aseptic failure group was older (*P* = 0.013) and less likely to have TKA (*P* < 0.001) (Table 2). The PJI group had a greater CRP (48.78 ± 84.71 vs. 4.25 ± 5.61 mg/L,* P* < 0.001), ESR (46.32 ± 29.99 vs. 14.49 ± 11.07 mm/h,* P *< 0.001), NLR (4.43 ± 2.98 vs. 2.61 ± 2.42,* P *< 0.001), PLR (220.84 ± 126.95 vs. 146.15 ± 57.07,* P* < 0.001), PVR (34.59 ± 12.72 vs. 27.33 ± 10.51,* P *= 0.001), GLB (36.28 ± 7.50 vs. 31.18 ± 5.26,* P* < 0.001), and CRP/AGR (51.32 ± 86.69 vs. 3.25 ± 4.18,* P* < 0.001). AGR in the PJI group was significantly lower than that in the aseptic failure group (*P* < 0.001) (Fig. 2 and Table 3).

**Table 2:** Basic characteristics and biomarkers in the PJI and aseptic failure groups

Characteristic	PJI (*N* = 47)	Aseptic failure (*N* = 117)	*P*
Age (years)	63.55 ± 11.56	68.44 ± 11.15	0.013*
BMI (kg/m^2^)	24.74 ± 3.44	24.62 ± 5.31	0.887
Gender			0.085
Male	22	38	
Female	25	79	
Affected joint			< 0.001*
Knee	28	21	
Hip	19	96	

Values are given as mean ± SD

*BMI* body mass index

*P < 0.05

**Table 3:** Values of the tested biomarkers in the PJI and aseptic failure groups

Biomarker	PJI (*N* = 47)	Aseptic failure (*N* = 117)	*P*
CRP (mg/L)	48.78 ± 84.71	4.25 ± 5.61	< 0.001*
ESR (mm/h)	46.32 ± 29.99	14.49 ± 11.07	< 0.001*
NLR	4.43 ± 2.98	2.61 ± 2.42	< 0.001*
PLR	220.84 ± 126.95	146.15 ± 57.07	< 0.001*
PVR	34.59 ± 12.72	27.33 ± 10.51	0.001*
GLB (g/L)	36.28 ± 7.50	31.18 ± 5.26	< 0.001*
AGR	1.11 ± 0.31	1.36 ± 0.24	< 0.001*
CRP/AGR	51.32 ± 86.69	3.25 ± 4.18	< 0.001*

Values are given as mean ± SD, *P < 0.05

*CRP* C-reactive protein, *ESR* erythrocyte sedimentation rate, *NLR* neutrophil-to-lymphocyte ratio, *PLR* platelet-to-lymphocyte ratio, *PVR* platelet-to-mean-platelet-volume ratio, *GLB* globulin, *AGR* albumin-to-globulin ratio

**Fig. 3:**
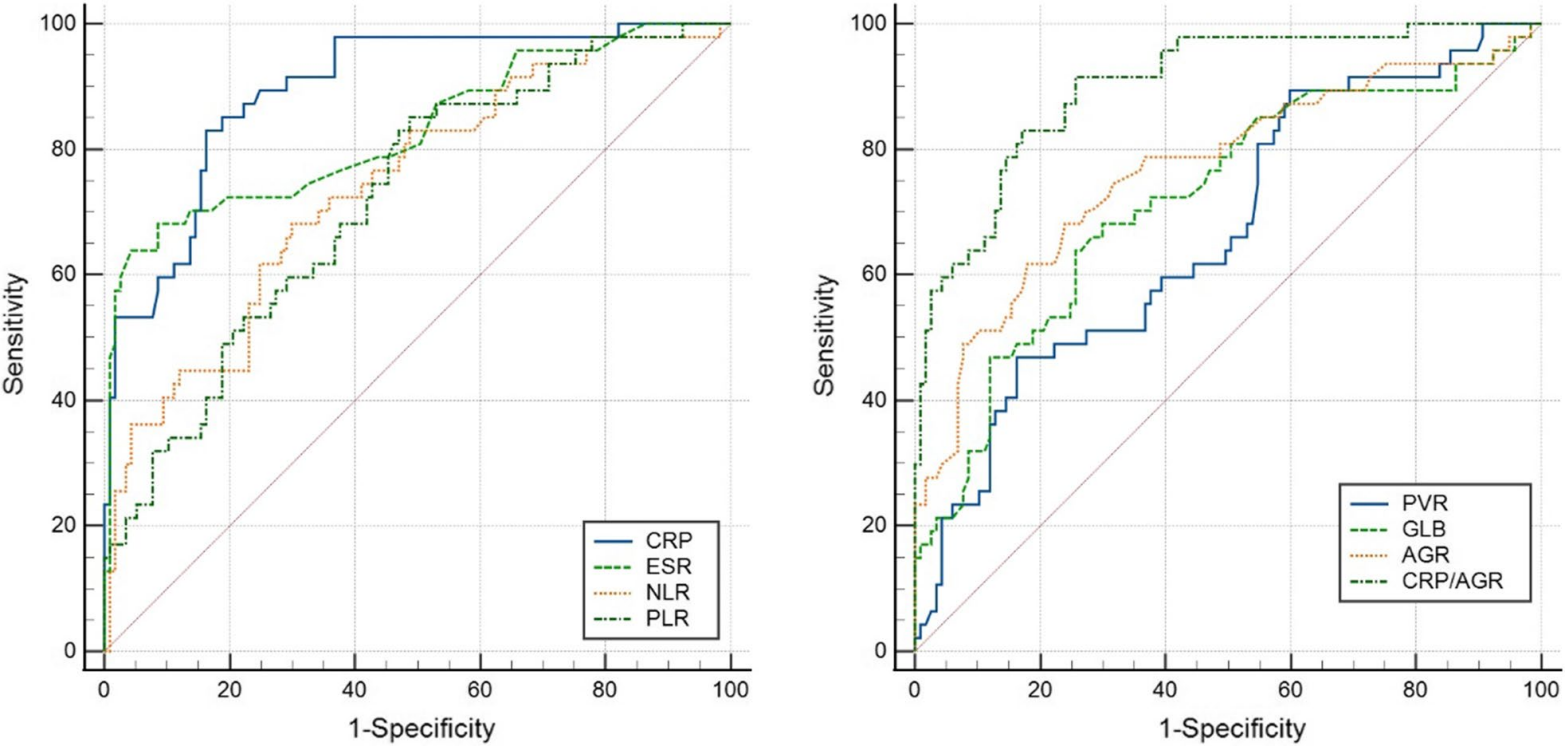
ROC curves for the diagnosis of PIJ based on NLR, PLR, PVR, GLB, AGR, and CRP/AGR relative to CRP and ESR (traditional biomarkers)

ROC analyses (Fig. 3 and Table 4) indicated that CRP/AGR performed best in diagnosing PJI (AUC: 0.902, 95% CI: 0.845–0.943; optimal cutoff: 5.08), followed by CRP (AUC: 0.896, 95% CI: 0.838–0.938; optimal cutoff: 6.59 mg/L) and ESR (AUC: 0.829, 95% CI: 0.763–0.883; optimal cutoff: 34 mm/h). The diagnostic performance levels of AGR (AUC: 0.767, 95% CI: 0.694–0.829; optimal cutoff: 1.19), NLR (AUC: 0.740, 95% CI: 0.666–0.805; optimal cutoff: 2.71), PLR (AUC: 0.721, 95% CI: 0.646–0.788; optimal cutoff: 132.67), and GLB (AUC: 0.719, 95% CI: 0.644–0.787; optimal cutoff: 33.8 g/L) were fair, and the diagnostic performance of PVR (AUC: 0.668; 95% CI: 0.590–0.739; optimal cutoff: 34.31) was poor.

**Table 4:** Performance of different individual biomarkers for the diagnosis of PJI

Biomarker	AUC (95%CI)	Optimal cutoff	Sensitivity	Specificity	PPV	NPV
CRP	0.896 (0.838–0.938)	6.59 mg/L	82.98%	83.76%	67.24%	92.45%
ESR	0.829 (0.763–0.883)	34 mm/h	63.83%	95.73%	85.72%	86.82%
NLR	0.740 (0.666–0.805)	2.71	68.09%	70.09%	47.77%	84.54%
PLR	0.721 (0.646–0.788)	132.67	85.11%	51.28%	41.24%	89.55%
PVR	0.668 (0.590–0.739)	34.31	46.81%	83.76%	53.66%	79.68%
GLB	0.719 (0.644–0.787)	33.8 g/L	63.83%	74.36%	50.00%	83.65%
AGR	0.767 (0.694–0.829)	1.19	68.09%	76.07%	53.34%	85.58%
CRP/AGR	0.902 (0.845–0.943)	5.08	82.98%	82.91%	66.11%	92.38%

*AUC* area under the curve,* PPV* positive predictive value,* NPV* negative predictive value

Our further analysis indicated that the optimal cutoff value was 2.71 for NLR, and this led to a sensitivity of 68.09%, specificity of 70.09%, positive predictive value (PPV) of 47.77%, and negative predictive value (NPV) of 84.54%. The optimal cutoff for PLR was 132.67 (sensitivity 85.11%, specificity 51.28%, PPV 41.24%, and NPV 89.55%); the optimal cutoff for PVR was 34.31 (sensitivity 46.81%, specificity 83.76%, PPV 53.66%, and NPV 79.68%). At the optimal cutoff value, the sensitivity, specificity, PPV, and NPV were 63.83%, 74.36%, 50.00%, 83.65% for GLB; 68.09%, 76.07%, 53.34%, 85.58% for AGR; and 82.98%, 82.91%, 66.11%, 92.38% for CRP/AGR, respectively. For the traditional biomarkers, the optimal cutoff for ESR was 34 mm/h with sensitivity: 63.83%, specificity: 95.73%, PPV: 85.72%, and NPV: 86.82%; the optimal cutoff for CRP was 6.59 mg/L with sensitivity: 82.98%, specificity: 83.76%, PPV: 67.24%, and NPV: 92.45% (Table 4).

According to the culture results and infection time, a subgroup analysis of patients in the PJI group was performed. Among the 47 PJI patients, 32 patients had positive culture results, whereas 15 had negative culture results. Based on the culture results, the most common pathogen was *Staphylococcus aureus* (31.25%, 10/32), followed by *Staphylococcus epidermidis* (12.50%, 4/32) (Table 5). Compared with the culture-negative PJI patients, the culture-positive PJI patients showed a significantly higher level of CRP (60.82 ± 96.38 vs. 23.08 ± 44.40 mg/L, *P* = 0.002), NLR (4.95 ± 3.20 vs. 3.33 ± 2.16, *P* = 0.035), PLR (234.04 ± 111.87 vs. 192.69 ± 154.90, *P* = 0.034), and CRP/AGR (62.97 ± 97.94 vs. 26.26 ± 48.49, *P* = 0.004). No significant differences were detected in the biomarkers ESR, PVR, GLB, and AGR between the culture-positive and culture-negative PJI patients (*P* > 0.05) (Table 6). Based on the optimal threshold value, the diagnostic accuracies for culture-positive PJI were CRP 93.75%, ESR 68.75%, NLR 78.13%, PLR 93.75%, PVR 50.00%, GLB 65.63%, AGR 78.13%, and CPR/AGR 93.75%. For culture-negative PJI, the diagnostic accuracies of CRP, ESR, NLR, PLR, PVR, GLB, AGR, and CPR/AGR were 60.00%, 53.33%, 46.67%, 66.67%, 40.00%, 60.00%, 46.67%, and 60.00%, respectively. Acute PJI was defined as an infection occurring within 90 days, and an infection after more than 90 days was regarded as chronic PJI. No significant difference was detected for the tested biomarkers between acute PJI and chronic PJI (Table 6).

**Table 5:** Culture results of patients in the PJI group (*n* = 47)

Culture result	Number of patients
Positive	32
* Staphylococcus aureus*	10
* Staphylococcus epidermidis*	4
*Staphylococcus hominis*	2
Methicillin-resistant* Staphylococcus epidermidis*	2
Methicillin-resistant* Staphylococcus aureus*	2
* Enterococcus faecium*	2
* Pseudomonas aeruginosa*	2
* Salmonella cholerae*	2
*Escherichia coli*	2
* Streptococcus lactis*	1
* Fengorella magna*	1
* Enterococcus faecalis*	1
* Proteus mirabilis*	1
Negative	15

**Table 6:** Comparison of all tested biomarkers in the different PJI subgroups

Biomarkers	Culture-positive PJI (*N* = 32)	Culture-negative PJI (*N* = 15)	*P*	Acute PJI (*N* = 10)	Chronic PJI (*N* = 37)	*P*
CRP (mg/L)	60.82 ± 96.38	23.08 ± 44.40	0.002*	70.08 ± 142.90	43.02 ± 62.49	0.459
ESR (mm/h)	48.94 ± 29.03	40.73 ± 32.24	0.373	45.00 ± 29.97	46.68 ± 30.40	0.845
NLR	4.95 ± 3.20	3.33 ± 2.16	0.035*	4.28 ± 4.23	4.47 ± 2.62	0.298
PLR	234.04 ± 111.87	192.69 ± 154.90	0.034*	172.75 ± 55.15	233.84 ± 137.90	0.363
PVR	34.99 ± 12.76	33.74 ± 13.04	0.741	34.03 ± 14.27	34.74 ± 12.48	0.755
GLB (g/L)	36.14 ± 7.15	36.59 ± 8.46	0.616	32.95 ± 6.48	37.18 ± 7.58	0.164
AGR	1.09 ± 0.33	1.14 ± 0.24	0.167	1.27 ± 0.36	1.06 ± 0.28	0.122
CRP/AGR	62.97 ± 97.94	26.26 ± 48.49	0.004*	68.77 ± 144.15	46.52 ± 65.23	0.311

Values are given as mean ± SD

*CRP* C-reactive protein, *ESR* erythrocyte sedimentation rate, *NLR* neutrophil-to-lymphocyte ratio, *PLR* platelet-to-lymphocyte ratio, *PVR* platelet-to-mean-platelet-volume ratio, *GLB* globulin, *AGR* albumin-to-globulin ratio

*P < 0.05

## Discussion

PJI is a very serious complication that can occur after total joint arthroplasty and has a devastating consequence if not diagnosed properly. Thus, the MSIS initially proposed criteria for the diagnosis of PJI and then modified these criteria during the International Consensus Meeting (ICM) in 2013 [6]. However, it is still difficult to diagnose PJI preoperatively in some cases, such as those with dry aspiration, negative culture findings, and systemic inflammatory diseases. In an effort to accurately diagnose PJI in a more timely manner, several previous studies examined the potential use of novel biomarkers such as CD4^+^ blood monocytes [19], α-defensin [20], leukocyte esterase [8], and calprotectin [21], and found that they had greater diagnostic value than traditional biomarkers, including CRP and ESR. However, the measurement of these novel parameters can be expensive and is unavailable in some institutions. Our purpose was to identify simple and practical biomarkers for the early diagnosis of PJI. Thus, we assessed the diagnostic performance of NLR, PLR, PVR, GLB, AGR, and CRP/AGR, the biomarkers that are easily obtained from routine blood tests, and then compared their diagnostic performance with those of the traditional biomarkers. To compare the diagnostic value of novel biomarkers, the ROC curves—which are used as a measure of the performance of a screening or diagnostic test—and the AUC values of these indicators were calculated. A higher AUC of a biomarker is usually associated with a higher diagnostic value for PJI.

Based on a literature review, we found that this study is the first to show that CRP/AGR gives a better diagnostic performance for PJI compared with traditional biomarkers (CRP and ESR). In this study, we compared the diagnostic performance of the tested biomarkers in terms of distinguishing patients in the PJI and the aseptic failure groups. Each of these novel biomarkers showed a significant difference in PJI patients compared with aseptic failure patients, similar to the traditional biomarkers. Our ROC analysis indicated that the optimal cutoff values of the novel biomarkers were 2.71 for NLR, 132.67 for PLR, 34.31 for PVR, 33.8 for GLB, 1.19 for AGR, and 5.08 for CRP/AGR. CRP/AGR was the only serum biomarker that showed excellent diagnostic performance, with a sensitivity of 82.98% and a specificity of 82.91%, followed by CRP and ESR, which showed good diagnostic performance. NLR, PLR, GLB, and AGR showed fair diagnostic performance, and PVR provided only poor diagnostic value for PJI. Notably, PLR had the highest sensitivity (85.11%) among all five tested biomarkers, and its sensitivity was even greater than those of CRP (82.98%) and ESR (63.83%).

Serum albumin is typically used as the biomarker to evaluate nutritional condition, which has been proven to be negatively related to septic failure after joint replacement [22]. Serum globulin mainly contains immunoglobulins, interleukin-6, and complements; it responds to infection and elevates with inflammatory reactions [23]. Thus, serum albumin, globulin, and the albumin-to globulin ratio (AGR) are utilized to determine a patient’s infective status, including PJI after joint replacement. In a prospective study by Huang et al., it was found that hypoproteinemia (albumin < 35 g/L) is closely related to aseptic failure after joint replacement [24]. A retrospective study by Shang et al. reported that the level of GLB was significantly elevated and the level of AGR was significantly decreased in a PJI group, with an AUC of 0.784 for GLB and 0.826 for AGR [18]. Another study by Shi et al. demonstrated that CRP/albumin gave excellent performance in the diagnosis of PJI, with an AUC of 0.941, which was better than those of CRP (0.937) and ESR (0.914) [25]. Inspired by the research of Shi et al., we used CRP/AGR as a novel biomarker to predict PJI, and found that this reduced the error associated with the use of either CRP or AGR to diagnose infection [25]. For the first time, we found that CRP/AGR gave better performance than CRP and ESR, with an AUC of 0.902.

PVR, NLR, and PLR are easily accessible and routinely available parameters in clinical practice. Besides the convenience and minimal expense necessary, previous studies have reported that these three biomarkers are generally useful for the diagnosis of infection [26]. A retrospective study by Paziuk et al. [13] demonstrated that the PVR provided acceptable performance for diagnosing PJI, with an AUC of 0.69 (48.10% sensitivity, 80.85% specificity) at a cutoff value of 31.70, similar to our cutoff value (34.31). They also reported that the combined application of PVR, CRP, and ESR significantly improved diagnostic performance. Sigmund et al. [16] evaluated the diagnostic value of PVR in a cohort of 177 patients who required revision surgery after THA (*n* = 91) and TKA (*n* = 86). They found that the individual use of PVR gave a sensitivity of 43% and a specificity of 81%, significantly inferior to those of CRP. They also found that the combined use of CRP + PVR did not improve the diagnostic performance relative to CRP alone. Our results are consistent with those of Sigmund et al. [16]. We therefore conclude that PVR should not be considered a reliable test for the diagnosis of PJI. Other studies also considered the use of NLR and PLR as biomarkers for inflammatory responses and infections. Qu et al. [27] measured the NLR in 2160 patients with bloodstream infections (BSIs) and 2523 healthy controls and found that NLR was significantly higher in the BSI group. They concluded that NLR had a strong association with BSIs caused by Gram-negative bacteria, Gram-positive bacteria, and fungi. Shen et al. [28] demonstrated that an elevated PLR was related to an increased risk of mortality, based on an analysis of the clinical data on 5537 patients with sepsis. Some other recent studies found that NLR and PLR were potentially useful for predicting PJI. In particular, a retrospective study by Zhao et al. [29] demonstrated that NLR and PLR were significantly higher in an early PJI group than in a non-PJI group, and that NLR might be more valuable than PLR based on ROC analysis. They used an NLR cutoff of 2.77, similar to our cutoff (2.71). Similarly, Yu et al. [14] found that NLR was effective in diagnosing PJI (AUC: 0.802, 85% sensitivity, 68.3% specificity, 34.7% PPV, 95.8% NPV) with a cutoff value of 2.13. However, Zhao et al. [29] and Yu et al. [14] demonstrated that NLR and PLR had greater predictive value for the diagnosis of PJI than ESR and CRP, in stark contrast to our results. Our further analysis indicated that this was most likely due to differences in the characteristics of the enrolled patients; in our study, all examined patients had chronic PJI, but the other two studies [14, 29] examined patients who had acute PJI. Thus, we conclude that NLR and PLR have only limited diagnostic value for PJI.

The pathogen culture result is the most valuable indicator of a diagnosis of PJI, and it can be used to guide the subsequent antibiotic selection. However, in some cases, due to a combination of microbial, host, and antibiotic factors, the microbiological culture results remain negative. According to the previous studies, the prevalence of culture-negative PJI ranges from 5 to 42% [30–32]. Thus, we conducted a subgroup analysis based on the culture results. In this study, the incidence of culture-negative PJI was 31.91%. We found that there were significant differences in terms of CRP, NLR, PLR, and CRP/AGR between the culture-positive PJI subgroup and the culture-negative PJI group, indicating that these biomarkers have the potential to predict negative culture results. However, all the tested biomarkers showed lower diagnostic accuracies in culture-negative PJI than in culture-positive PJI. Thus, more attention should be paid to the diagnosis of culture-negative PJI. Some novel strategies, such as a delayed incubation period of up to 14 days, utilizing augmented media for atypical organisms, sonication-based and chemical-based biofilm dislodgment methods, and next-generation sequencing technologies, have been recommended to improve the yield of the culture [33].

There are several limitations of our study. First, this is a retrospective study and therefore has the limitations inherent to studies with this design. Our exclusion of patients with missing critical data or complicated by autoimmune diseases might have led to some bias. Second, there is no gold standard for the diagnosis of PJI. However, the MSIS criteria are considered the best method for its diagnosis, although this standard has low sensitivity in patients with low-virulence bacterial infections [19, 34]. To reduce the possibility of misdiagnosis, we excluded patients who underwent second-stage reimplantation due to the difficulty of determining their infection status. Finally, we examined 164 cases from a single institution, and this small sample size limited the generalizability of our conclusions. Therefore, well-designed multicenter studies with larger samples are needed to evaluate the value of novel biomarkers for the diagnosis of PJI.

## Conclusion

In conclusion, we found that the levels of NLR, PLR, PVR, GLB, AGR, and CRP/AGR were significantly higher in patients diagnosed with PJI, and these biomarkers may therefore have potential for the diagnosis of PJI. However, when used alone, only CRP/AGR showed excellent performance in the diagnosis of PJI, followed by CRP and ESR, with good diagnostic performance. NLR, PLR, GLB, and AGR showed fair diagnostic performance, and PVR showed only poor diagnostic value for PJI. Therefore, we conclude that CRP/AGR is a valuable test for diagnosing PJI, but that other novel biomarkers have only limited diagnostic value.

## Data Availability

According to the policy of our hospital, the raw data could not be shared with others without permission. An anonymized form of the data could be made available from the corresponding author upon reasonable request.

## Abbreviations

NLR: Neutrophil-to-lymphocyte ratio
PLR: Platelet-to-lymphocyte ratio
PVR: Platelet–to-mean-platelet-volume ratio
CRP: C-reactive protein
ESR: Erythrocyte sedimentation rate
ALB: Albumin
GLB: Globulin
AGR: Albumin-to-globulin ratio
MPV: Mean platelet volume
PJI: Periprosthetic joint infection
ROC: Receiver operating characteristic
AUC: Area under the curve
TKA: Total knee arthroplasty
THA: Total hip arthroplasty
MSIS: Musculoskeletal Infection Society
BMI: Body mass index
PPV: Positive predictive value
NPV: Negative predictive value
BSI: Bloodstream infections
TJA: Total joint arthoplasty
